# Identification and analysis of the FAD gene family in walnuts (*Juglans regia* L.) based on transcriptome data

**DOI:** 10.1186/s12864-020-6692-z

**Published:** 2020-04-15

**Authors:** Kai Liu, Shugang Zhao, Shuang Wang, Hongxia Wang, Zhihua Zhang

**Affiliations:** 1 Mountainous Area Research Institute of Hebei Province, Hebei Agricultural University, Baoding, 071001 China; 2 College of Life Sciences, Hebei Agricultural University, Baoding, 071001 China; 3 Research Center for Agricultural Engineering Technology of Mountain District of Hebei, Baoding, 071001 China; 4 National Engineering Research Center for Agriculture in Northern Mountainous Areas, Baoding, 071001 China

**Keywords:** Walnut, Fatty acid, Gene expression, *FAD* gene family, *FAD3*

## Abstract

**Background:**

Walnut kernels contain a large amount of unsaturated fatty acids, such as linoleic acid and linolenic acid, which are essential fatty acids for humans and have important effects on growth and health. The main function of fatty acid desaturase (FAD), which is widely distributed in organisms, is to remove hydrogen from carbon chains in the biosynthesis of unsaturated fatty acids to generate C=C bonds.

**Results:**

By performing a series of bioinformatics analysis, 24 members of the *JrFAD* gene family were identified from the genome database of walnut, and then compared with the homologous genes from *Arabidopsis*. Phylogenetic analysis showed that JrFADs were classified into four subfamilies: the SAD desaturase subfamily, Δ7/Δ9 desaturase subfamily, Δ12/ω-3 desaturase subfamily and “front-end” desaturase subfamily. Meanwhile, the expression of fatty acid synthesis genes in walnut kernels at different developmental stages was analysed by transcriptome sequencing, with expression of *JrFAD3-1*, which encodes an enzyme involved in linolenic acid synthesis, being particularly prominent. The relative expression level of *JrFAD3-1* changed dramatically with the kernel development stages and exhibited a Bell-Shaped Curve. A significant positive correlation was observed between the expression of *JrFAD3-1* during 70–100 DAF (Days after flowering) and the content of alpha-linolenic acid during 100–130 DAF, with a correlation coefficient of 0.991. Additionally, *JrFAD3-1* was proved closely related to homologous genes in *Betula pendula* and *Corylus heterophylla*, indicating that the conserved structure of FADs is consistent with classical plant taxonomy.

**Conclusion:**

Twenty-four members JrFADs in walnut were identified and classified into four subfamilies. *JrFAD3-1* may play significant roles in the biosynthesis of polyunsaturated fatty acids in walnut.

## Background

Walnut (*Juglans regia* L.) is an important economic tree species throughout the world and is widely cultivated as a traditional wood and oil crop. The walnut kernel oil content can be as high as 63%, higher than that of other leading oil crops, such as peanut, soybean, palm, olive, corn (as the germ), and sunflower [1]. Fatty acids, which are the main nutritional component accumulated in walnut kernels, comprise a large amount of unsaturated fatty acids, such as linoleic acid and linolenic acid [2], which are essential fatty acids for humans and have an important role in growth and health [3]. In addition, polyunsaturated fatty acids are prodrugs of prostaglandins, which have blood pressure-lowering, platelet adhesion-reducing, and anticoagulant effects [4]. However, the human body cannot synthesize linoleic acid and α-linolenic acid, which can only be obtained through the diet [5]. The unsaturated fatty acid content of walnut kernel oil is as high as 90% [3], which can inhibit the absorption of cholesterol in the small intestine and the inhibit the re-absorption of bile acid, promote the degradation and elimination of cholesterol in the liver, alter the distribution of cholesterol in the body, and accelerate the transfer of plasma cholesterol to vascular tissues [4].

Fatty acid desaturase (FAD) is widely present in organisms [6–9]. The main function of FAD is to remove hydrogen from carbon chains in the biosynthesis of unsaturated fatty acids to produce C=C bonds. In plants, FADs can be classified into two categories according to their solubility: soluble desaturases and membrane-integrins [10]. The stearoyl-ACP-desaturase (SAD) unique to higher plants is the only known FAD present in the matrix of plastids. All other types of desaturases belong to the integrin class and are localized to endoplasmic reticulum (FAD2 and FAD3) and plastid (e.g., FAD6, FAD7 and FAD8) membranes. The first step in the synthesis of unsaturated fatty acids is the introduction of a double bond at the Δ9 position [11], and the enzymes that catalyse the reaction include stearoyl-CoA-desaturase (SCD) [12] and SAD [13]. SAD, with the help of ferredoxin, uses the stearoyl carrier protein (stearoyl-ACP) to remove two hydrogens to form oleoyl ACP [14]. The second unsaturated bond is added by Δ12 desaturases (FAD2 and FAD6) based on the introduction of the first unsaturated bond [15]. Δ15 desaturases, such as FAD3, FAD7 and FAD8, play a key role in further decreasing the degree of saturation [16–18]. In particular, microsomal ω-3 fatty acid desaturase, one of the FAD3 catalysed the synthesis of linolenic acid, is located in the endoplasmic reticulum and uses phospholipids as acyl substrates, and NADH, NADH-cytochrome *b*_*5*_ reductase and Cyt *b*_*5*_ as electron donors [19].

The enzyme encoded by the *FAD3* gene is a member of the ω-3 fatty acid dehydrogenase family, a membrane integrin distributed in the endoplasmic reticulum that catalyses the introduction of a third unsaturated bond. The FAD3 amino acid sequence is highly conserved with three histidine-rich conserved domains, which together with divalent iron ions constitute the reaction centre of fatty acid dehydrogenase [20]. The Lys-Lys-X-X (KKXX) recovery signal contained at the C-terminus of the amino acid sequence is consistent with the properties of endoplasmic reticulum integrins [20]. Since the first *FAD3* was isolated from *Arabidopsis* [21], researchers have successively cloned genes from plants such as rapeseed, perilla, peony and *Eucommia ulmoides*. In recent years, the functional regulation of *FAD3* in the synthesis and metabolism of unsaturated fatty acids has become a hot research topic.

With the release of walnut genome data, genes involved in the metabolism of walnut nutrients have been discovered. Indeed, a number of key genes encoding enzymes involved in walnut fatty acid metabolism, such as ACCase [22–24], have been cloned and characterized. In this research, walnut kernels at different stages of maturity were used as test materials to analyse the key gene family involved in unsaturated fatty acid synthesis based on bioinformatics analysis and transcriptome sequencing. This study explored the mechanism of walnut fatty acid formation, especially the anabolic mechanism of unsaturated fatty acids, and the results provide a theoretical basis for the regulation of walnut fatty acid synthesis. Furthermore, this study offers a theoretical reference for the rational development of walnut oil and oil-producing walnut cultivars.

## Results

### Identification and analysis of FAD genes in the walnut genome

We identified the FAD family using *Arabidopsis* FAD family protein sequence information to construct the hidden Markov model. The walnut protein data were searched, and 33 FAD family genes were screened. Using Pfam domain analysis, 24 FAD family genes were ultimately obtained, encoding 30 protein sequences, which are numbered according to their annotation in walnut (Table 1).

**Table 1:** Basic information of the FAD family in walnut

Name	Locus ID	NCBI Reference	Gene length (bp)	Exon number	Intron number	CDS (bp)	Amino acid length (AA)	PI	MW (KD)
JrADS3	LOC108998330	XM_018974856.1	2497	5	4	1164	387	9.95	44.56
JrALD	LOC109021249	XM_019003847.1	2055	1	0	1344	447	8.56	51.34
JrDAL	LOC109001694	XM_018979084.1	4240	1	0	1149	382	8.78	44.11
JrDES1-1	LOC109011517	XM_018992767.1	2103	2	1	981	326	7.27	37.91
JrDES1-2	LOC108990634	XM_018964647.1	2623	2	1	978	325	7.83	37.74
JrFAD2-1	LOC109021930	XM_019004668.1	3197	1	0	1167	388	8.72	44.38
JrFAD2-2	LOC109021930	XM_019004667.1	3411	1	0	1167	388	8.72	44.38
JrFAD2-3	LOC109011954	XM_018993369.1	6737	1	0	1152	383	8.83	43.90
JrFAD2-4	LOC109011954	XM_018993367.1	4273	1	0	1152	383	8.83	43.90
JrFAD3-1	LOC109002248	XM_018979912.1	2402	8	7	1143	380	8.26	43.88
JrFAD3-2	LOC108989905	XM_018963672.1	2166	7	6	1053	350	7.79	40.51
JrFAD3-3	LOC108989905	XM_018963671.1	2166	8	7	1143	380	7.37	43.61
JrFAD3-4	LOC108989903	XM_018963670.1	2129	7	6	1077	358	7.80	41.43
JrFAD3-5	LOC108989903	XM_018963669.1	2128	8	7	1167	388	7.39	44.50
JrFAD4	LOC108983937	XM_018955731.1	1262	1	0	924	307	9.11	34.68
JrFAD6	LOC108993197	XM_018968019.1	4917	10	9	1329	442	9.18	51.38
JrFAD7	LOC109007160	XM_018986723.1	3038	8	7	1368	455	8.94	52.06
JrFAD8-1	LOC108994930	XM_018970364.1	2963	8	7	1368	455	8.98	51.77
JrFAD8-2	LOC109015144	XM_018997622.1	2965	8	7	1368	455	8.98	51.77
JrSAD-1	LOC109005061	XM_018983828.1	5401	3	2	1191	396	6.44	45.26
JrSAD-2	LOC108984606	XM_018956614.1	5286	3	2	1191	396	6.39	45.16
JrSAD-3	LOC108984606	XM_018956616.1	5039	2	1	1074	357	5.72	40.99
JrSAD-4	LOC108984606	XM_018956617.1	2456	2	1	999	332	5.40	38.05
JrSAD-5	LOC108988959	XM_018962389.1	2413	3	2	1176	391	7.72	44.43
JrSAD6	LOC109012153	XM_018993662.1	1549	2	1	1185	394	5.98	44.63
JrSAD-6	LOC108989429	XM_018963029.1	2075	3	2	1206	401	8.71	45.49
JrSAD-7	LOC108988967	XM_018962401.1	1981	3	2	1170	389	8.26	44.32
JrSAD-8	LOC109018931	XM_019001129.1	14,280	4	3	1002	333	6.16	38.08
JrSLD-1	LOC109009165	XM_018989549.1	1905	1	0	1344	447	6.64	32.64
JrSLD-2	LOC109002707	XM_018980564.1	1846	1	0	1344	447	6.42	32.48

Because of the limited depth of analysis of walnut genome data, the specific distribution of these genes on the 16 pairs of chromosomes in walnut is unclear. The shortest length among these *FAD* genes is 1262 bp, and the longest length is 14,280 bp. The CDS lengths of these genes ranged from 924 bp-1368 bp. The amino acids sequence length of FADs ranged varied from 307 to 455 amino acids (aa). The predicted molecular weight (MW) of these proteins ranged from 32.48 kDa (JrSLD-2) to 52.06 kDa (JrFAD7), and the theoretical isoelectric points (pI) ranged from 5.40 to 9.95.

The length of *JrFAD3-1* is 2402 bp, including 8 exons and 7 introns; the coding region is 1143 bp, encoding 380 amino acids.

### Analyses of the evolution, exon-intron structure and motif distribution of JrFAD family members

**Fig. 1:**
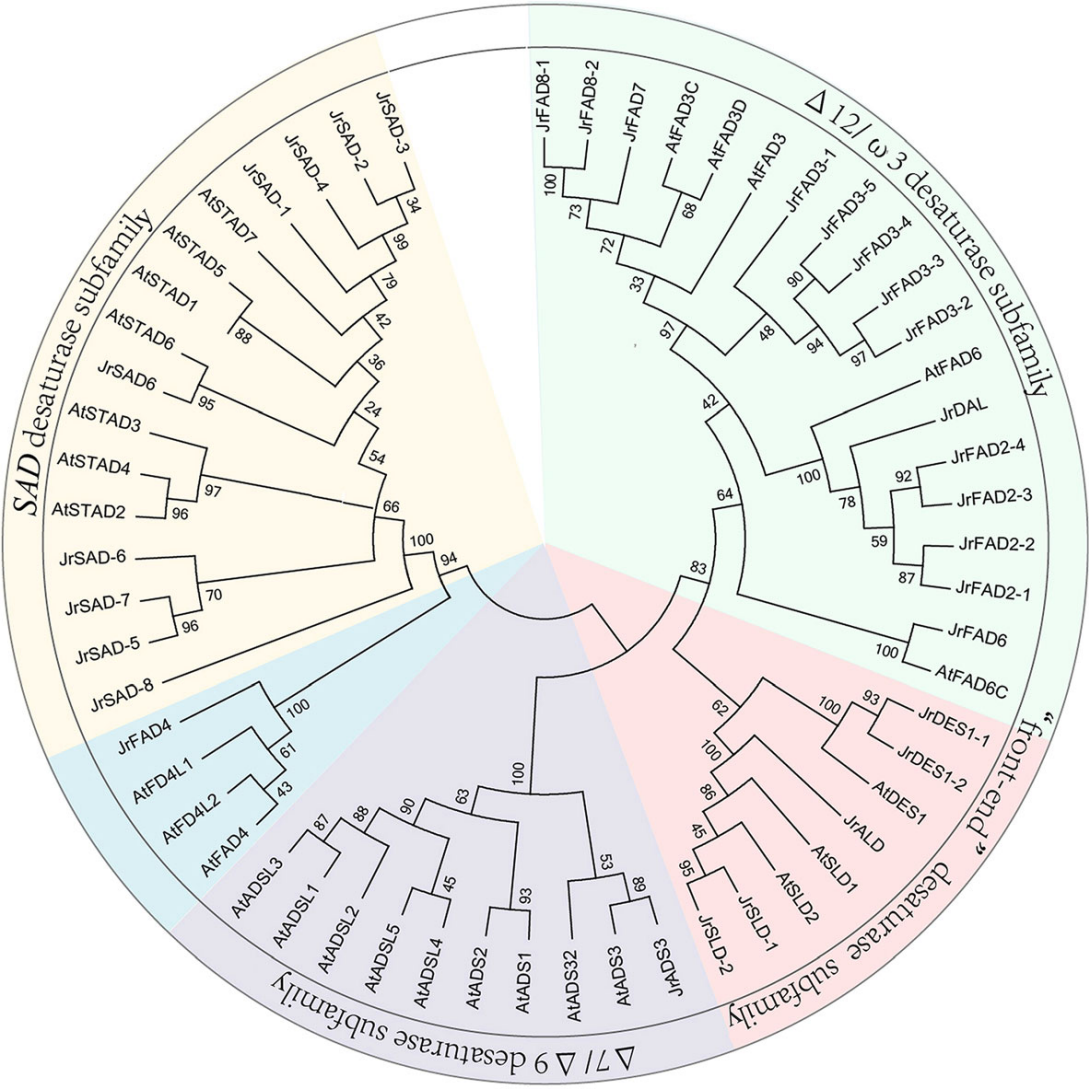
Phylogenetic analysis of JrFADs (*Juglans regia*) and AtFADs (*Arabidopsis thaliana*) with a total of 57 protein sequences. MEGA 5.0 was used to construct the phylogenetic tree employing the maximum likelihood (ML) method. A total of 1000 bootstrap replications were carried out to indicate reliability

Using MEGA 5.0 software with the maximum likelihood (ML) method, the walnut FADs protein sequences were constructed together with the *Arabidopsis* FADs protein sequences to build a phylogenetic tree (Fig. 1), indicating that the FAD gene families of *J. regia* and *A. thaliana* are similar. There are four main subfamilies: the SAD desaturase subfamily, Δ7/Δ9 desaturase subfamily, Δ12/ω-3 desaturase subfamily and “front-end” desaturase subfamily.

**Fig. 2:**
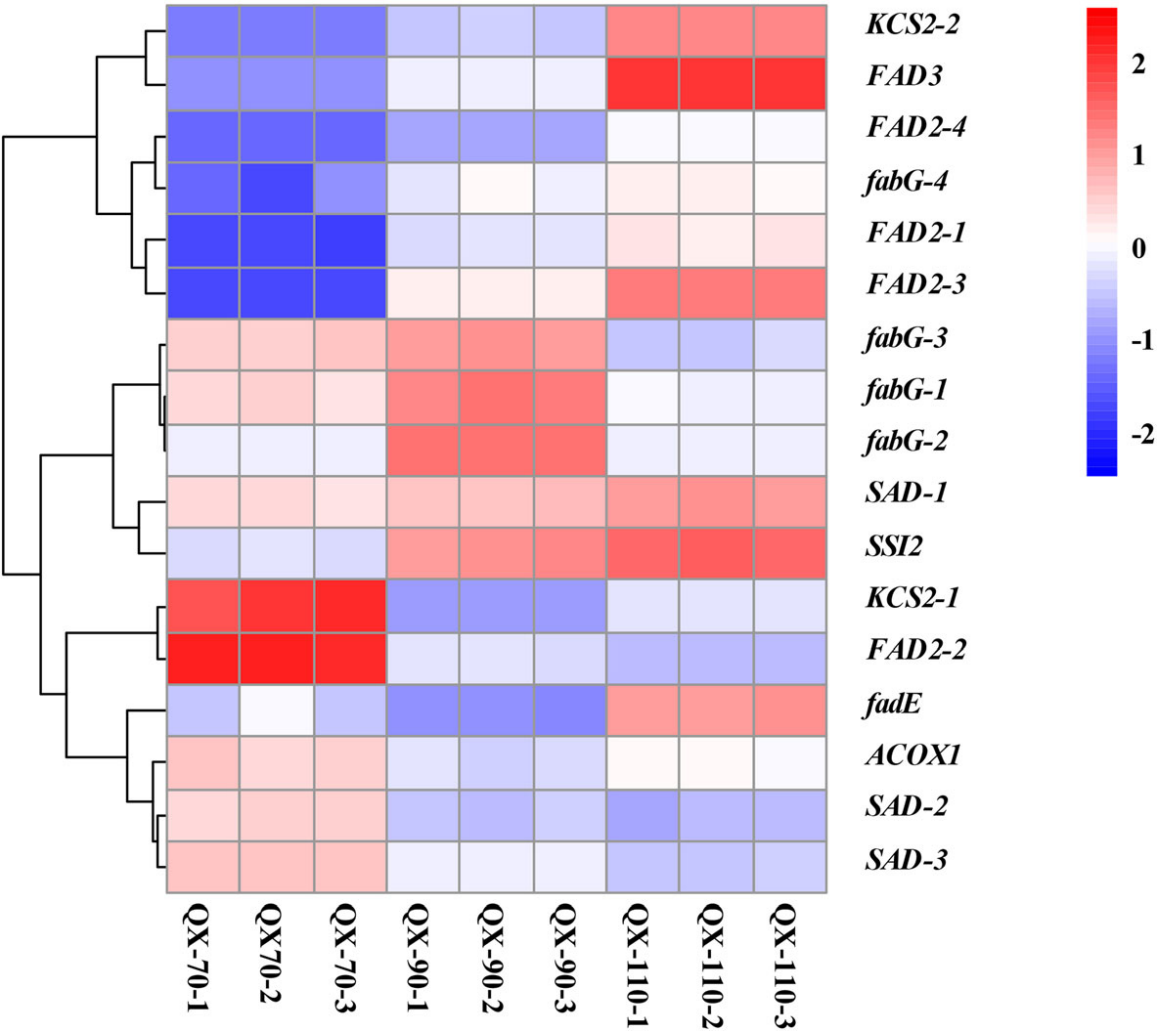
Expression profilles (heatmaps) of genes associated with unsaturated fatty acid biosynthesis. Red indicates high gene expression level; white indicates medium level and blue indicates low level

To date, SAD is the only subfamily of soluble enzymes in the FAD family, and the remaining types of fatty acid desaturases are membrane integrins [25, 26]. There are 7 copies of the *SAD* gene in *Arabidopsis* and 9 in walnut, and these genes are well clustered in the unified phylogenetic tree. In the Δ12/ω-3 desaturase subfamily, six branches of the walnut ω-6 desaturase and two from *Arabidopsis* grouped together as Δ12 fatty acid desaturases. Three *Arabidopsis FAD3* genes and eight annotated ω-3 desaturase genes in walnuts together form the Δ12/ω-3 desaturase subfamily; among the five *FAD3* genes of walnut, *FAD3-1* was found to be far from the other four in evolutionary distance. Based on a comparison of gene expression levels during the development of walnut kernels (Fig. 2 and Additional file 1 Table S1), expression of *FAD3* was strongly detected in developing kernels; the other four genes were not expressed or expressed at very low levels, indicating that *FAD3-1* is the key enzyme gene that catalyses the synthesis of linolenic acid in walnut kernels. A similar expression profile was reported for *FAD* genes in olive, and siRNA was able to suppress the expression of other *FADs* but not *FAD2-3* [27].

**Fig. 3:**
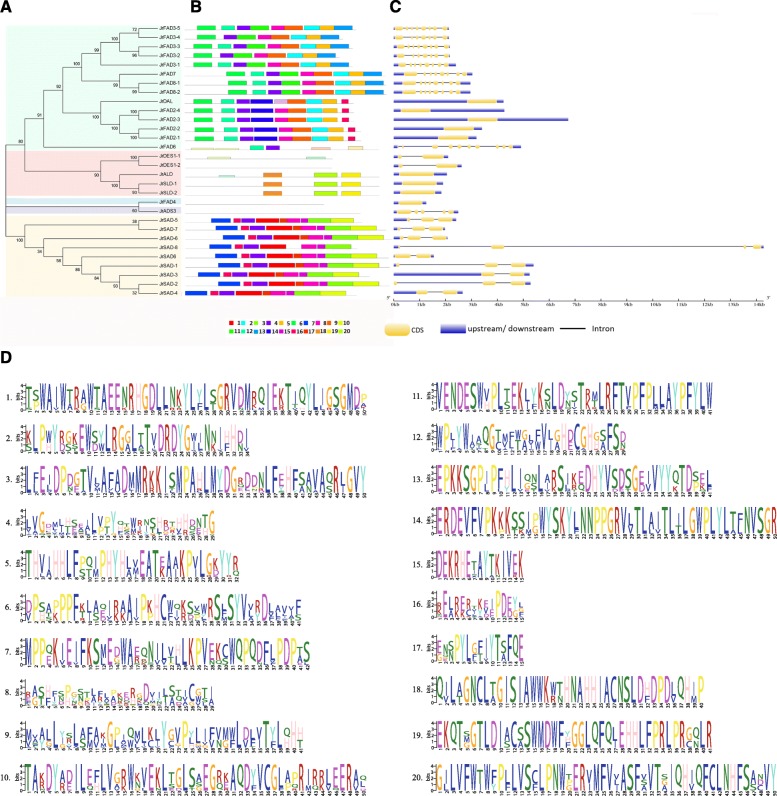
Gene structure and conserved motif analysis of the FAD family from *Juglans regia*. **a** Phylogenetic analysis of JrFAD family; **b** Conserved motif of JrFAD family; **c** Gene structure of JrFAD family; **d** Motifs of walnut FAD family proteins. The motifs in the JrFADs were identified using Multiple Em for Motif Elicitation (MEME). In JrFADs proteins, 20 conserved motifs were identified and shown in different colors

To further investigate the structural evolution of walnut FADs, we first analysed the exon-intron structure (Fig. 3). The FAD genes contain 1–9 introns except *JrALD*, *JrSLD-1*, *JrSLD-2*, *JrDAL*, *JrFAD2-1*, *JrFAD2-2*, *JrFAD2-3*, *JrFAD2-4* and *JrFAD4*. Moreover, genes in the same subfamily have similar intron and exon structures. *JrFAD6* differs from other Δ12 desaturase genes in that it has the largest number of introns and exons. Both *JrDES1* genes contain two exons, whereas *JrALD* and *JrSLDs* genes have only one exon. The number of exons in the SAD subfamily range between 2 and 4. *JrSAD-8* contains 4 exons, the highest number; while *JrSAD6*, *JrSAD-3* and *JrSAD-4* contain 2 exons, and the remaining genes contain 3 exons (Fig. 3c).

Subsequently, we used MEME software to analyse the conservation of these protein sequences and structures (Fig. 3b). Most of the 20 conserved motifs (Fig. 3d) found belong to the typical transmembrane helix region and unknown functional complex region in fatty acid desaturases. Although no common conserved motifs were observed among the 30 members of the walnut FAD family, the distribution of conserved motifs was very similar within the same subfamily. In the SAD subfamily, all genes except *JrSAD-8* contain conserved motifs1, − 3, − 4, − 7 and − 10; in the Δ12/ω-3 subfamily, the genes contain conserved motifs2, − 4, − 5, − 6, − 8 and − 9 except *JrFAD6*.

### Expression of genes related to unsaturated fatty acid synthesis

A heap map of our RNA-seq data highlighted differential expression of important metabolic pathways genes at kernel development stages, which showed the expression levels of lipid metabolism related genes were higher at late stage (Additional file 2 Fig. S1; Additional file 3 Table S2). The expression of genes related to unsaturated fatty acid biosynthesis and arachidonic acid metabolism peaked at 110 DAF based on the number of reads by transcriptome sequencing. The expression of genes related to alpha-linolenic acid metabolism first decreased and then increased, and reaching the maximum value at 110 DAF, based on the number of reads by transcriptome sequencing (Additional file 4 Fig. S2; Additional file 5 Table S3).

Seventeen genes enriched in the metabolic pathway of unsaturated fatty acid biosynthesis were selected (Fig. 2). *KCS2* (encoding 3-ketoacyl-CoA thiolase), and *fabG* (encoding 3-oxoacyl-ACP reductase) are involved in carbon chain elongation; *fadE* (encoding acyl-ACP desaturase), *SSI2* (encoding acyl-ACP desaturase), *FAD2* (encoding fatty acid desaturase 2), *FAD3-1*(encoding fatty acid desaturase 3), *ACOX1* (encoding peroxisomal acyl-CoA oxidase) and *SAD* (encoding stearoyl-ACP-desaturase) are involved in the desaturation process*.* The observed expression of 17 transcripts of these 8 genes can be roughly divided into two categories. On the one hand, the expression level was lower at 70 DAF but higher at 110 DAF. There were 7 transcripts, *fabG-4*, *KCS2-2*, *fadE*, *FAD2-1*, *FAD2-3*, *FAD2-4* and *FAD3-1*, which were mainly involved in the biosynthesis of linoleic acid, linolenic acid and isounsaturated fatty acids. On the other hand, the expression levels were relatively high at 70 DAF and then gradually decreased. Ten transcripts, *fabG-1*, *fabG-2*, *fabG-3*, *SAD-1*, *SAD-2*, *SAD-3*, *SSI2*, *ACOX1*, *KCS2-1* and *FAD2-2*, mainly participate in the biosynthesis of oleic acid and linoleic acid. Expression of *FAD3-1* increased rapidly during the period from 70 DAF to 110 DAF. The dehydrogenation of linoleic acid to α- linolenic acid began at 70 DAF, though the α-linolenic acid content was almost zero, but genes encoding enzymes that catalyze the dehydrogenation of linoleic acid to linolenic acid were highly expressed at this stage. With the rapid increase in the expression of *FAD3-1*, the content of α-linolenic acid in the kernel began to increase gradually.

### Tissue-specific expression of *JrFAD* family genes

**Fig. 4:**
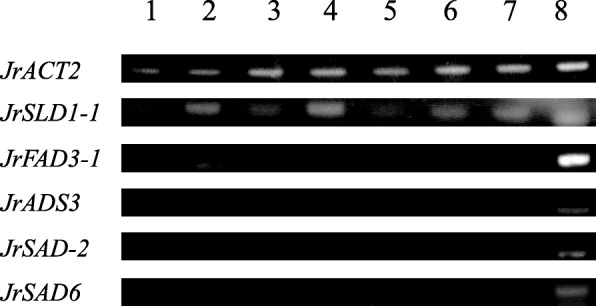
Expression pattern of *JrFAD* genes in eight tissues/organs by RT-PCR. *JrACT2* was used as an internal control. From left to right: pistils, catkin, young stem, older stem, young leaves, mature leaves, young embryo, and mature embryo

By semi-quantitative detection (Fig. 4), *SLD-1* was found to be expressed in all 8 tissues, among which the expression levels were higher in catkins, old branches, mature leaves and kernels. *DAL* was most highly expressed in young and mature leaves. *JrFAD3-1* was expressed in catkins, young leaves and kernels, and the highest expression level was observed in mature embryos. *ADS3*, *SAD-2* and *SAD6* were only expressed in mature embryos. It can be preliminarily concluded that the FAD family is characterized by the desaturase subfamily and that the Δ7/Δ9 desaturase subfamily is specifically expressed in the embryo but that the “front-end” desaturase subfamily is expressed in all tissues. The Δ12/ω-3 desaturase subfamily is highly expressed in mature embryos. However, the determination of more specific expression patterns still requires further research and verification.

### Expression of JrFAD3-1 and accumulation of α-linolenic acid in kernels at different developmental stages

**Fig. 5:**
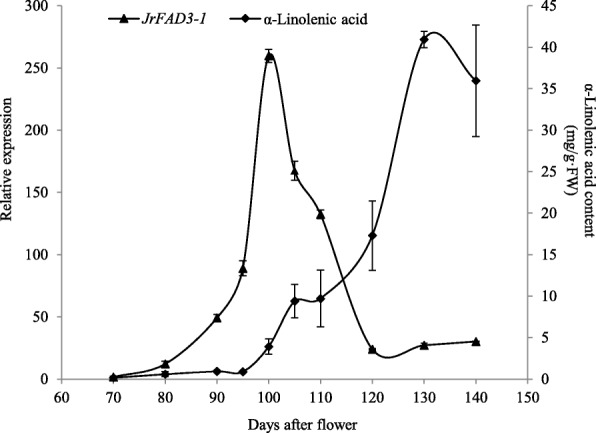
Relative expression of *JrFAD3-1* gene and α-Linolenic acid content in the kernels at different developmental stages

The relative expression of *JrFAD3-1* increased slowly at 70 DAF in the ‘Qingxiang’ kernel, increasing rapidly after 90 DAF and peaking at 100 DAF (Fig. 5). Then, its expression quickly decreased and gradually stabilized at a lower level after 120 DAF. We also detected the content of α-linolenic acid in identical samples and found that it was maintained at a low level from 70 DAF to 95 DAF and gradually accumulated from 95 DAF to 120 DAF. The content increased rapidly from 120 DAF to 130 DAF and peaked at 40.92 mg/g, after which it decreased slightly (Fig. 5). Combining the two results revealed a 30-day difference between the peak of *JrFAD3-1* gene expression and that of α-linolenic acid content. Expression of *JrFAD3-1* at 70–100 DAF and content of α-linolenic acid at 100–130 DAF was also analysed, showing a significant positive correlation between expression of *JrFAD3-1* at 5 periods (70–100 DAF) and the content of α-linolenic acid at 5 periods (100–130 DAF), with a correlation coefficient of 0.991. Normally, the kernel water content decreased during the late fruit development [1]. Therefore, it is possible that the decrease in *JrFAD3-1* expression and the decrease in water content in the late stage are factors contributing to the increase in the α-linolenic acid content.

### Multiple sequence alignment of FAD3 proteins

Multiple sequence alignment of the FAD3 proteins among 32 species was performed using MEGA 5.0. The results showed that FAD3 proteins were conserved between monocotyledonous and dicotyledonous plants and yeast. Additionally, conserved domain prediction was performed by a database search of the Pfam protein domain family, and all FAD3 proteins of the 32 species contain the fatty acid desaturase motif. Through comparative analysis, 62 completely conserved sites in the 33 protein sequences were found (Additional file 6 Fig. S3). It can be further confirmed that the primary structure of the JrFAD3-1 protein is closely related to the content of α-linolenic acid.

### Phylogenetic evolution of FAD3 proteins

**Fig. 6:**
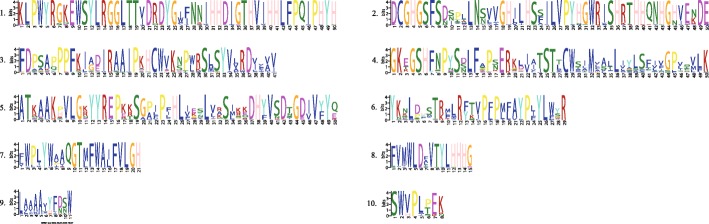
Conserved motifs of the FAD3 proteins in 32 species

**Fig. 7:**
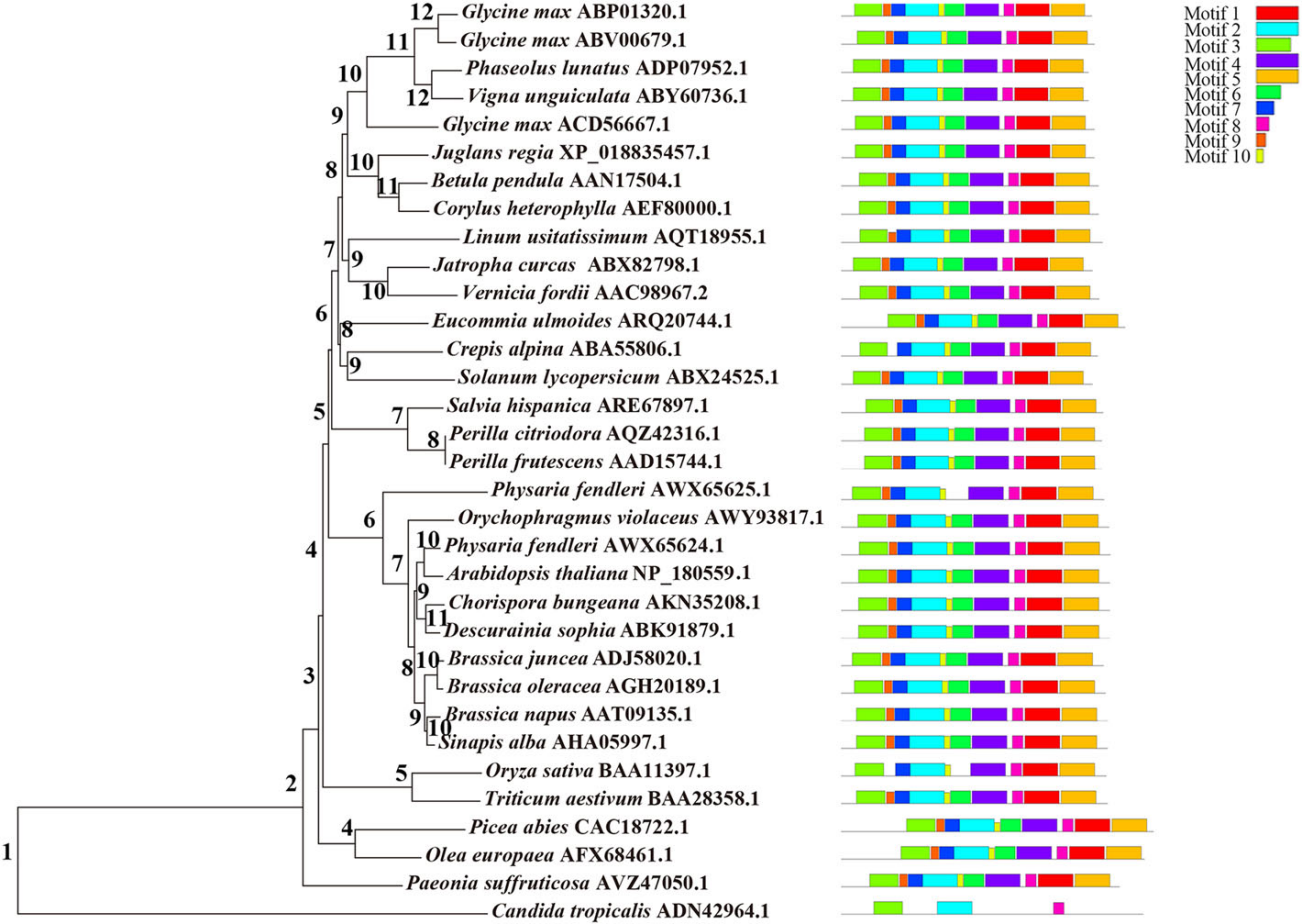
Phylogeny conserved motif of FAD3 proteins in 32 species. Number represents branch series. The motifs in the FAD3 proteins were identified using MEME. Ten conserved motifs were identified of 32 species and shown in different colors

Using MEGA 5.0, the retrieved homologous proteins were assessed along with the JrFAD3-1 protein and the reported sequences from the 32 species mentioned above. Subsequently, we used MEME software to analyse conservation among these protein sequences. Most of the 10 conserved motifs (Fig. 6) belong to the typical transmembrane helix region and unknown functional complex region in fatty acid desaturases. Three common conserved motifs (motif 2, motif 3 and motif 8) were found among the 33 FAD3 proteins of the 32 species, but the distribution of conserved motifs was very similar within the same branch. The 32 species are divided into 2 branches (Fig. 7). *Candida tropicalis* is in one group with only 3 motifs (motif 2, motif 3, motif 8), and the other 31 plants are in another group. Of the latter, the 31 species are divided into 2 branches. *Paeonia suffruticosa* is a kind of flower or oil crop, and it grouped alone; the other plants clustered in another group. *Picea abies* and *Olea europaea* also group together though they are evolutionarily far, and the other comprise another group. The remaining 28 species are divided into 2 groups, 2 monocots (*Oryza sativa* and *Triticum aestivum*) and 26 dicots (e.g., *Glycine max*, *Phaseolus lunatus*, *J. regia*). Except for *Crepis alpina*, *Physaria fendleri*, and *O. sativa*, all FADs of 28 higher plants contain conserved motifs 1–10; the FAD3 of *O. sativa* contains 8 conserved motifs except motif 6 and motif 9. JrFAD3-1 is closely related to homologous genes of *Betula pendula* (85.75%) and *Corylus heterophylla* (84.92%). *B. pendula*, *C. heterophylla* and *J. regia* all belong to the order Fagales, which further indicates that the conserved structure of FADs is consistent with classical plant taxonomy.

## Discussion

To date, the FAD gene family has been identified and characterized in many plants, including 17 FADs in *Arabidopsis* [21], 29 in soybean [28], 31 in peanut [29], and 41 in Raymond cotton [30]. *FAD* genes have also been isolated from land cotton [31], sesame [32], sunflower [33] and other plants. In this study, 24 FAD family members were identified and analysed based on protein properties. In addition, a similar genetic structure was observed for each small branch, with high subfamily conservation. For example, the Δ12/ω-3 desaturase subfamily contains 7–8 exons, consistent with *Arabidopsis* [21] and *Carya cathayensis* [34]. There are 2–3 exons in SAD subfamily genes, consistent with peanut [29]. Studies have shown that the origin of introns is ancient and that insertion of introns is the result of exon rearrangement, with an important role in gene evolution [35, 36]. By analysing gene sequences, the *JrFAD* family was found to be highly conserved, though there are large differences between subfamilies. The FAD family of walnut and *Arabidopsis* is composed of four major groups: the SAD desaturase subfamily, the Δ7/Δ9 desaturase subfamily, the Δ12/ω-3 desaturase subfamily and the “front-end” desaturase subfamily [21]. There are large differences in the copy number of genes in each subfamily. The Δ9 desaturase subfamily in *Arabidopsis* has the most common gene copy number of 9 [21], whereas the gene copy number is only one in walnut. Similar to the results for walnut, Δ9 desaturase was not identified in the genome of soybean; instead, *SAD* [28], which also generates the first unsaturated bond as dose Δ9 desaturase, was detected. This suggests thatΔ9 desaturase, which is widely distributed among organisms, is replaced by the SAD subfamily in higher plant, with similar functions and greater copy numbers, which may be related to the evolution of the *SAD* gene family in plants [21]. The number of Δ12/ω-3 desaturase subfamily members is also higher in walnut than in *Arabidopsis*, probably because these genes have not undergone related genome-wide replication during evolution.

There is accumulating evidence that the linolenic acid content is closely related to the *FAD3* gene [37–40]. In *Arabidopsis* seeds, specific overexpression of endogenous *FAD3* increased the α-linolenic acid content from 19 to 40% [41], and overexpression of *FAD3* genes in tomato resulted in significantly increased α-linolenic acid levels in the leaves and fruits of the transgenic plants [42]. Ni et al. [43] reported that the content of α-linolenic acid in rapeseed increased when *BnFAD3* was overexpressed. Inhibition of tobacco *FAD3* gene expression through RNAi technology significantly reduced the accumulation of α-linolenic acid [44]. Liao et al. [45] suppressed the expression of *FAD2*, *FAD3* and *FATB* in rapeseed by constructing a multi-gene interference vector containing the seed-specific promoter NapinA, increasing the oleic acid content by more than 16%. The content of linolenic acid in walnut kernels was high, and 5 copies of *JrFADs* were identified in the genome-wide range.

Although 5 *FAD3* copies were identified from the walnut genome, only *JrFAD3-1* expression was found in the walnut kernel during development, with the peak expression at approximately 100 DAF. Although *JrFAD3-1* is not a seed-specific gene, expression of this gene was significantly upregulated during seed development, showing a trend of increase and then decrease, with a tendency toward a decline as the seed matures, consistent with the findings of previous studies [46, 47]. This change may be related to the important role of *FAD3* in the synthesis of unsaturated fatty acids in walnut [48]. There may be two reasons for the change in α-linolenic acid content after 30 days in this study. First, a process in the cytoplasm may be involved. Proteins are involved in mRNA production and must be transported from the cytoplasm to the nucleus after translation; there is also a time lag in gene expression due to such processed as post-translational modification [49, 50]. Second, the α-linolenic acid content is directly related to the amount of jasmonic acid produced; ω-3 fatty acid dehydrogenase (FAD) catalyses the synthesis of α-linolenic acid from linoleic acid [51]. *JrFAD3-1* encodes Δ15 fatty acid desaturase in the seed, catalysing the conversion of linoleoyl CoA to α-linoleyl CoA. α-Linolenic acid is the substrate for jasmonic acid [52]. The protein content reached its highest level at 110 DAF, showing a positive correlation with the oil content [53]. It is also possible that the α-linolenic acid content due to a lack of substrate in the early stage was not obvious early in embryo development in walnut. As the embryo matured, expression of *JrFAD3-1* was downregulated, and the α-linolenic acid content increased. Overall, desaturation gradually decreased after expression peaked, which may have been caused by the decrease in FAD3 activity and the consumption of α-linoleic acid. In general, gene function can be explained comprehensively through positive and negative aspects, providing a theoretical basis for establishing a complete metabolic network.

## Conclusions

This study described the FAD gene family of walnut at the genome level. Their gene structure, phylogenetic relationship, and tissue-specific expression patterns were presented in this study. A total number of 24 members of the *JrFAD* gene family were identified and classified into four major subfamilis. *JrFAD3-1*, a key gene in Δ12/ω-3 desaturase subfamily, was obtained based on transcriptome data, and its expression was analysed. The function of *JrFAD3-1* was also characterized based on the deduced phylogeny. The result predicates that *JrFAD3-1* may play a key role in the biosynthesis of polyunsaturated fatty acids. This study lays the foundation for further functional elucidation of JrFAD genes in walnut.

## Methods

### Plant materials

The material used in this study was collected from the Experimental Field of Hebei Agricultural University in 2016. A walnut cultivar of ‘Qingxiang’ in full fruit-bearing period was selected as the test material.

The samples used for transcriptome sequencing were ‘Qingxiang’ kernels collected 70 (QX-70), 90 (QX-90) and 110 (QX-110) DAF. Nine samples of walnut kernels at each stage were collected from ‘Qingxiang’ trees. Several grams of different tissues from each plant were frozen in liquid nitrogen and stored at − 80 °C.

*JrFAD3-1* gene expression analysis was conducted with ‘Qingxiang’ samples from 10 different developmental stages (70 DAF, 80 DAF, 90 DAF, 95 DAF, 100 DAF, 105 DAF, 110 DAF, 120 DAF, 130 DAF and 140 DAF), and 8 different tissues: pistils, catkins, young branches (bark), old branches (bark), young leaves, mature leaves, young embryos and mature embryos. After the fruits were collected, the embryos were quickly removed by peeling and immersed in liquid nitrogen, and stored at − 80 °C for RNA extraction, expression analysis and determination of fatty acid content.

The kits used for RNA extraction and DNA extraction were from Tiangen (product numbers: DP441 and DP350 respectively). The reagents used for reverse transcription, PCR and real-time PCR were from Takara (product numbers: RR047A, RR901A and RR820A, respectively).

### Cloning of the JrFAD3-1 gene

The *JrFAD3-1* gene was amplified from kernels collected 100 DAF. The *JrFAD3-1* partial gene sequence was identified from walnut genome data, and a homology-based cloning method was used to isolate the *JrFAD3-1* coding sequence (CDS) from ‘Qingxiang’ walnut according to the gene sequences of soybeans and castor beans. Premier 5.0 software was used to design specific primers as follows: *JrFAD3*-*1*-F: 5’- GAGAAAGGAGGAGAGAATGC-3’, *JrFAD3*-1-R: 5’- GATAGCCTTGCT. CTTCAAA-3’.

### Identification of *FAD* gene family and bioinformatics analysis

The hidden Markov model was constructed using HMMER 3.1b software and the *Arabidopsis* FAD family protein sequence (Additional file 7 Table S4) in the UniProt database and the protein sequences of walnut were downloaded from GenBank (accession GCF_001411555.1). The conserved domains of the proteins were analysed using the Pfam protein family database (http://pfam.org/) [54] of the European Institute of Bioinformatics, using the website GSDS (Gene Structure Display Server: http://gsds.cbi.pku.edu.cn/) [55] and MEME (http://meme-suite.org/) pair for the walnut FAD family. The phylogenetic tree of JrFAD and AtFAD gene family was constructed using the maximum likelihood method in MEGA 5.0 software with a bootstrap value of 1000. The phylogenetic tree JrFAD and 31 other species were built using the maximum likelihood method in MEGA 5.0 software with a bootstrap value of 1000.

### Analysis of transcriptome sequencing data

The predicted coding transcripts were aligned with the GenBank NR (https://www.ncbi.nlm.nih.gov/nucleotide/), Swiss-Prot (https://www.uniprot.org/), InterPro (http://www.ebi.ac.uk/interpro/), GO (http://geneontology.org), and KEGG databases (https://www.kegg.jp/) for functional annotation (Additional file 8).

### Tissue-specific expression in the JrFAD family

For RNA isolation experiments, all samples were immediately frozen in liquid nitrogen and extracted using a Tiangen Plant RNA Extraction Kit according to the manufacturer’s protocol. First-strand cDNA was synthesized with a Tiangen Inverse Transcription Kit. The gene-specific primers used for RT-PCR and qRT-PCR were designed using NCBI (Table 2). Five genes belonging to four subfamilies were selected to analyse the expression of the FAD families in different tissues.

**Table 2:** Primers sequences of *JrFADs* and *JrACT2* for qRT-PCR

Name	Sequence (5′–3′)	
*JrACT2*	F: TCCACCATGTTCCCTGGTAT;	R: ACCTCCCAATCCAGACACTG
*JrSLD-1*	F: ACTTGGTTTGGGAGGCTGTC;	R: TCGTCCTAACAACCCCAAGC
*JrADS3*	F: TTCCTTGTGTGGGGAATGGG;	R: AAATGCAAGCAATGCCACCC
*JrSAD-2*	F: TTTTTCCCCTTCTCAACCTCCAA;	R: TGGTGTAAAAGGCTTCTTTGGATTG
*JrSAD6*	F: AAGCTGGAGGATTTGACGGG;	R: CCAGCTAAACTTAACGCCGC
*JrFAD3-1*	F: ATCCACCATGATATCGGCACC;	R: GAAGGGGAATTGGCCCAGA

The PCR procedure was as follows: 94 °C for 5 min; 33 cycles of 94 °C for 30 s, 54 °C for 30 s, and 72 °C for 1 min; and 72 °C for 10 min.

### Gene expression analysis by quantitative RT-PCR

qRT-PCR experiments were performed using an FX96 Touch™ Real-Time PCR Detection System (Bio-Rad) with a SYBR Green I Master Kit (Tiangen). *JrACT2* (NCBI Reference: XM_018972062.1) was used as the reference gene to calculate relative fold differences based on the comparative cycle threshold (2^-∆∆ct^). The PCR mixture (20 μL) contained 10 μL SYBR-Green PCR Master Mix (Tiangen), 0.2 μM each primer, 100 ng cDNA template, and nuclease-free water. The PCR procedure was as follows: 95 °C for 5 min; 30 cycles of 94 °C for 10 s, 60 °C for 30 s, and 72 °C for 30 s; followed by a final extension at 72 °C for 3 min.

### Determination of fatty acid content

The determination of unsaturated fatty acids referred to Bai [56].

### Statistical analysis

Data were analysed using SPSS 17.0 software.

## Supplementary information

**Additional file 1 **: **Table S1** Expression data of genes associated with unsaturated fatty acid biosynthesis.

**Additional file 2 **: **Fig. S1** Clustering analysis (heatmap) of annotations for metabolic pathways.

**Additional file 3 **: **Table S2** Expression data of the key metabolic pathways genes at kernel development stages.

**Additional file 4 **: **Fig. S2** Expression of fatty acid biosynthesis metabolic pathway-related genes at different development stages.

**Additional file 5 **: **Table S3** Expression of genes related to unsaturated fatty acid biosynthesis and arachidonic acid metabolism.

**Additional file 6 **: **Fig. S3** Multiple conserved sequence alignment of FAD3 proteins in 32 species.

**Additional file 7 **: **Table S4** Information of the FAD family in *Arabidopsis thaliana*.

**Additional file 8.** Annotation genes sequences of transcriptome data.

## Data Availability

All data and materials are presented in the main paper and additional file. In addition, the whole protein sequences of FADs in *Arabidopsis* were retrieved from TAIR databases. The CDS and genome sequences of FADs in walnut were retrieved from the whole walnut genome database (accession GCF_001411555.1) in NCBI.

## Abbreviations

FAD2: Microsomal Δ12 desaturase
FAD3: Microsomal ω-3 desaturase
FAD4: Trans Δ3 desaturase
FAD5: Δ7 desaturase
FAD6: Plastidial Δ12 desaturase
FAD7: Plastidial ω-3 desaturase
FAD8: Plastidial ω3 desaturase
ADS: Δ9 desaturase
SAD: Stearyl acyl-carrier-protein desaturase
ALD: Acyl-lipid (9-3)-desaturase
SCD: Stearoyl-CoA-desaturase
ACCase: Acetyl coenzyme A carboxylase
KCS2: 3-ketoacyl-CoA thiolase
fabG: 3-oxoacyl-ACP reductase
fadE: Acyl-CoA dehydrogenase
ACP: Acyl-carrier protein
DAL: Δ12-acyl-lipid-desaturase
DES1: Sphingolipid Δ4-desaturase
SSI: Acyl-ACP desaturase
ACOX: Peroxisomal acyl-CoA oxidase
SLD: Δ8-desaturase
*Jr*: *Juglans regia*
