# Supplementary material for: Identification and analysis of the FAD gene family in walnuts (Juglans regia L.) based on transcriptome data
Source: BMC Genomics. 2020 Apr 15;21:299. doi: 10.1186/s12864-020-6692-z (PMC7158092; doi:10.1186/s12864-020-6692-z)
Supplement: Supplementary file 2 — Additional file 2 : Fig. S1 Clustering analysis (heatmap) of annotations for metabolic pathways. [file 12864_2020_6692_MOESM2_ESM.docx]

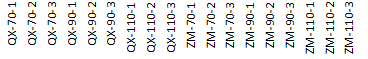

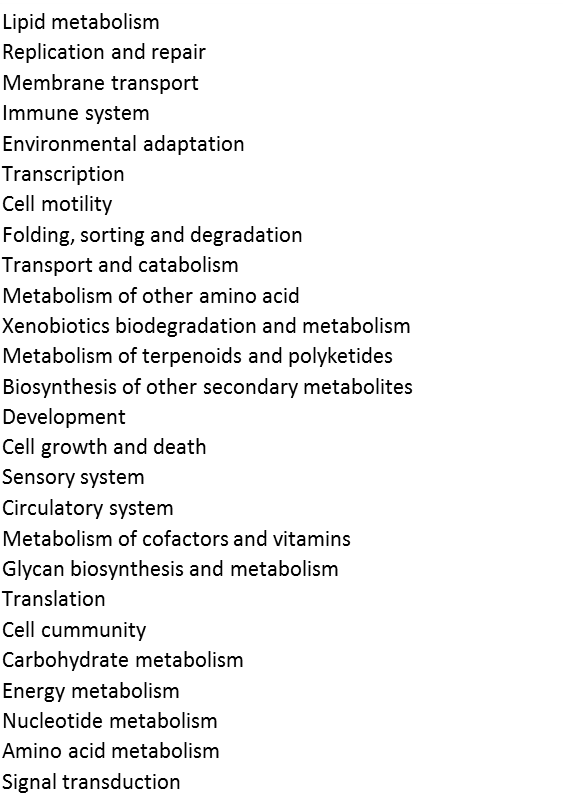


Figure S1 Clustering analysis (heatmap) of annotations for metabolic pathways. Red indicates high gene expression level; white indicates medium level and blue indicates low activity. KEGG categories were labled in the right of each heatmap line.
