# Supplementary material for: Identification and analysis of the FAD gene family in walnuts (Juglans regia L.) based on transcriptome data
Source: BMC Genomics. 2020 Apr 15;21:299. doi: 10.1186/s12864-020-6692-z (PMC7158092; doi:10.1186/s12864-020-6692-z)
Supplement: Supplementary file 4 — Additional file 4 : Fig. S2 Expression of fatty acid biosynthesis metabolic pathway-related genes at different development stages. [file 12864_2020_6692_MOESM4_ESM.docx]

Figure S2 Expression of fatty acid biosynthesis metabolic pathway-related genes at different development stages. FPKM: Fragments per kilobase million
