# Supplementary material for: Identification and analysis of the FAD gene family in walnuts (Juglans regia L.) based on transcriptome data
Source: BMC Genomics. 2020 Apr 15;21:299. doi: 10.1186/s12864-020-6692-z (PMC7158092; doi:10.1186/s12864-020-6692-z)
Supplement: Supplementary file 6 — Additional file 6 : Fig. S3 Multiple conserved sequence alignment of FAD3 proteins in 32 species. [file 12864_2020_6692_MOESM6_ESM.docx]

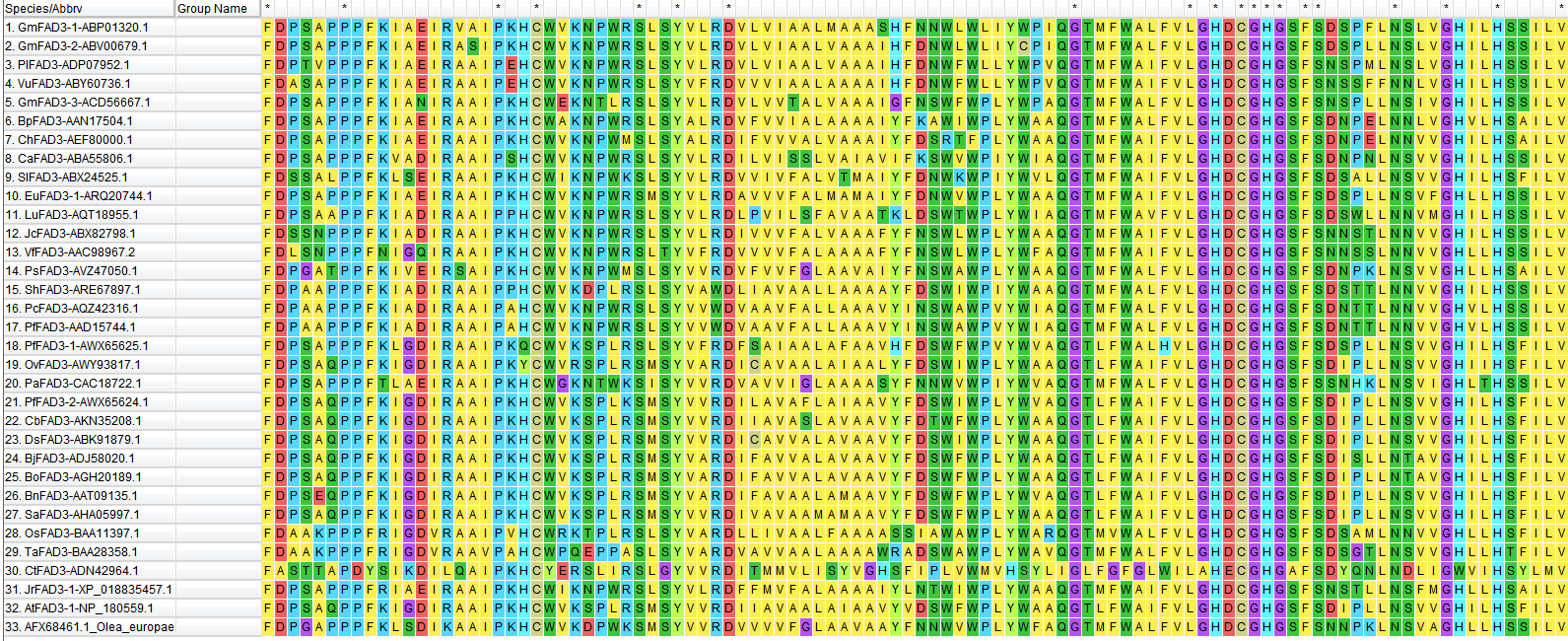


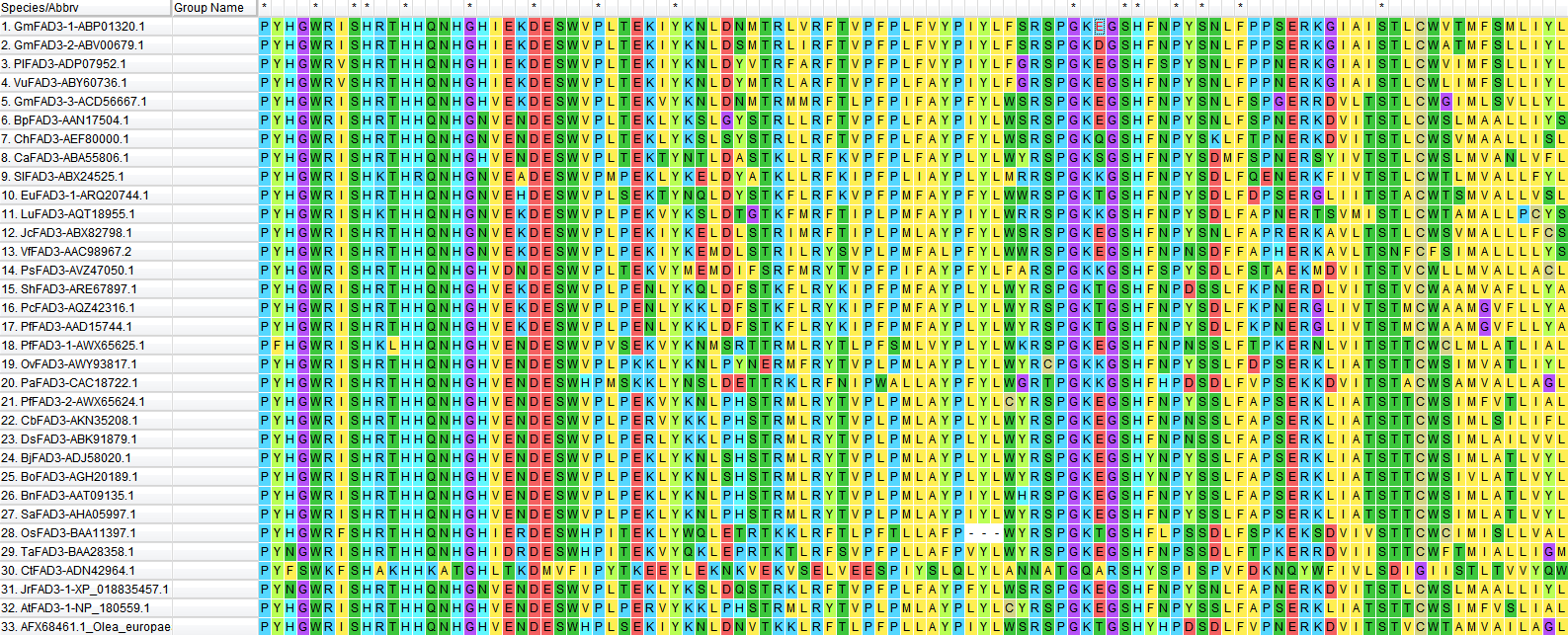


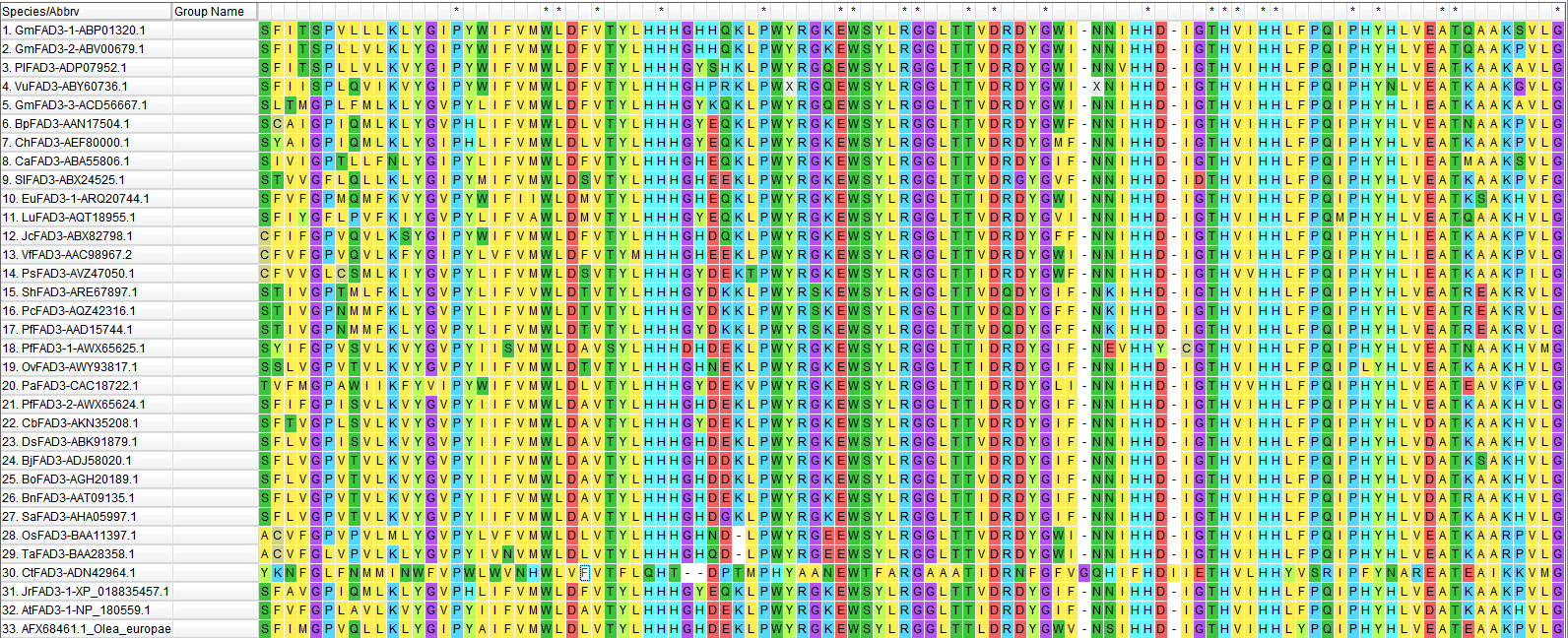


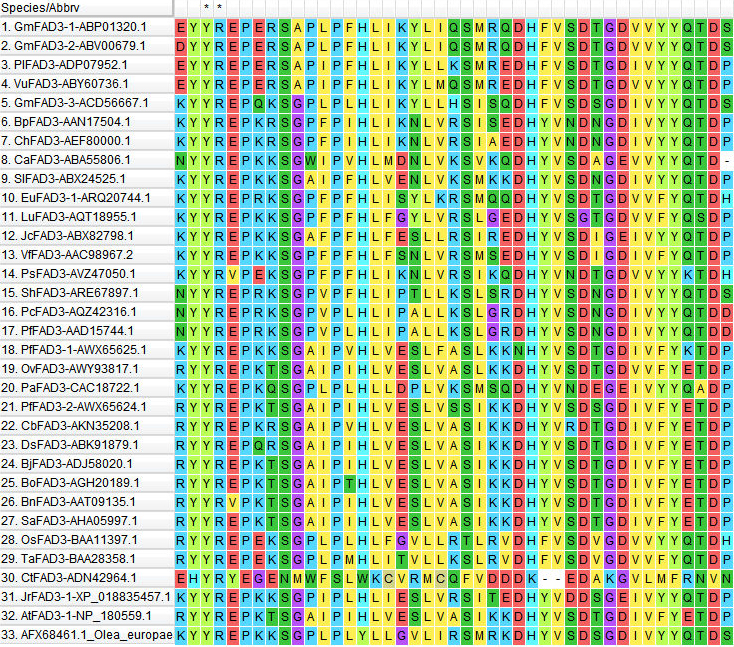


Figure S3 Multiple conserved sequence alignment of FAD3 proteins in 32 species

Note: dicot monocots gymnosperm fungi
